# Prevalence and Associated Factors of Fecal Incontinence and Double Incontinence among Rural Elderly in North China

**DOI:** 10.3390/ijerph17239105

**Published:** 2020-12-06

**Authors:** Yan Luo, Kai Wang, Ping Zou, Xiaomei Li, Jinjie He, Jing Wang

**Affiliations:** 1 Faculty of Nursing, Health Science Center, Xi’an Jiaotong University, 76# Yanta West Road, Xi’an 710061, China; luoyan0904@xjtu.edu.cn (Y.L.); roselee@xjtu.edu.cn (X.L.); hjj521209@stu.xjtu.edu.cn (J.H.); 2 Department of Epidemiology and Biostatistics, School of Public Health, Tongji Medical College, Huazhong University of Science and Technology, 13# Hang Kong Road, Wuhan 430030, China; kay_wang@hust.edu.cn; 3 School of Nursing, Nipissing University, 750 Dundas West, Room 209, Toronto, ON M6J 3S3, Canada; pingz@nipissingu.ca

**Keywords:** double incontinence, fecal incontinence, prevalence, correlate, rural China

## Abstract

Fecal and double incontinence are known to be more prevalent among the rural elderly. Yet, there have been few studies on their epidemic condition among Chinese rural elders. This study estimated the prevalence and correlates of fecal and double incontinence in rural elderly aged 65 years and over in North China. A multisite cross-sectional survey was conducted in 10 villages, yielding a sampling frame of 1250 residents. Fecal and urinary incontinence assessments were based on the self-reported bowel health questionnaire and the International Consultation on Incontinence Questionnaire-Short Form, respectively. The concomitant presence of fecal and urinary incontinence in the same subject was defined as double incontinence. The prevalence of fecal and double incontinence was 12.3% and 9.3%, respectively. Factors associated with fecal incontinence included urinary incontinence, lack of social interaction, traumatic brain injury, cerebrovascular disease, and poverty. Physical activities of daily living dependence, traumatic brain injury, lack of social interaction, and poor sleep quality were associated with higher odds of having double incontinence, whereas tea consumption was correlated with lower odds. Individualized intervention programs should be developed targeting associated factors and high-risk populations. These intervention programs should be integrated into existing public health services for the rural elderly to facilitate appropriate prevention and management of incontinence.

## 1. Introduction

Fecal incontinence (FI), the involuntary loss of solid and/or liquid stools, and double incontinence (DI), the concomitant presence of urinary and fecal incontinence in the same subject, are distressing health problems that are increasingly prevalent but underreported [1]. Two systematic reviews have reported a wide prevalence range of FI among community-dwelling adults (1.4–20.7%), largely because of the variability in the definition of FI used and population samples used [2,3]. The prevalence rate of DI among community-dwelling adults ranges from 1.7% to 24% and differs according to race and ethnicity [4,5,6,7,8]. Patients are often reluctant to report the problem and to seek treatment for incontinence due to feelings of embarrassment or the misconception that incontinence is a normal consequence of aging that cannot be treated [9]. Even when it occurs episodically, incontinence has embarrassing consequences on self-confidence, personal image, and mental ability [6,9]. Older adults with incontinence are also prone to dependency, frailty, increased caregiver burdens, and economic cost, leading to a substantial decrease in the quality of life [1]. Incontinence, especially DI, is reported as one of the leading causes of institutionalization and long-term hospitalization among older people [10]. In certain cases, this problem causes greater impairment than some chronic illnesses such as diabetes or arterial hypertension [2,3,4]. 

Studies conducted in high-income countries have demonstrated that incontinence can be prevented by addressing modifiable risk factors through primary prevention in the community or nursing homes [11,12]. Recognized risk factors for both urinary and fecal incontinence can be categorized into physical status (e.g., increasing age, obesity, constipation, limited physical activity, and cognitive impairment), psychosocial (e.g., changes in lifestyle, depression, and imagined or actual rejection by relatives), and environmental (e.g., inadequate lighting and heating in the toilet, lack of easy access to a toilet, and unsuitable clothing) [3,13,14]. Age-related changes, such as chronic diseases, cognitive impairment, and decline in daily activity, make older adults more vulnerable to incontinence.

In recent decades, many published studies have focused on the epidemiology of urinary incontinence among adults, especially women [15,16], but FI and DI among elders in low- and middle-income countries (LMICs), including China, remains an understudied topic [2,3]. A review of the prevalence and risk factors of incontinence noted that only three studies about FI have been carried out in developing countries, including FI among rural women aged 30 to 75 years in EI Salvador and anal incontinence among patients in hospitals in southeastern Nigeria and Sri Lanka [17]. The most recent large-scale epidemiologic survey of FI in mainland China was conducted between 2014 and 2015 among 28,196 women aged 20 years and older living in urban areas and indicated an overall prevalence of FI at 0.43% [18]. Another research conducted in an outpatient clinic and gynecology clinic of Taiwan reported a FI prevalence of 9.3% for women older than 65 years [19]. Considering lower health literacy, higher odds of dementia and functional disability, and the very limited healthcare resources among rural residents, studies on the prevalence and risk factors of incontinence in rural areas are more crucial than in urban areas [20,21]. The lack of long-term care service and traditional filial piety have made home-based informal care the dominant form of care in China and other Asian LMICs [22,23]. Therefore, the prevention and management of incontinence for the elderly in home settings are more crucial in these countries. This study aimed to explore the prevalence rate and associated factors of FI and DI in community-dwelling older people in rural areas of North China. The study is the baseline of a longitudinal cohort study, which aims to deeply explore the risk factors of incontinence in rural areas. 

## 2. Methods

### 2.1. Study Design and Population

From June 2017 to September 2017, a multisite cross-sectional study was conducted to explore the prevalence and correlates of FI and DI among older adults aged 65 years and over in rural China. A pilot study with 50 participants was conducted to estimate the sample size and to ensure the measurements were suitable and the training process for investigators were effective. The sample size for the present study was calculated based on a prevalence (p) of fecal incontinence of 12.0% among adults aged 65 years or older in the pilot study, the design effect (deff) of 2 (it was assumed that 100 residents would be selected from each village, ρ = 0.01, deff = $1+\left(n_{j}-1\right)\times \rho$ = 1.99), a confidence level of 95%, margin of error of 0.05 (Z_α/2_ = 1.96), an absolute error (d) of 3%, and a nonresponse rate of 10% using the formula *N* = $\mathrm{deff}\times Z_{\alpha /2}^{2}P\left(1-P\right)/d^{2}\times \left(1+10\%\right)$. We estimated a required sample size of approximately 992 subjects and 10 villages (*n* = 992/100 = 10). A cluster sampling procedure was used, and 10 villages were randomly selected based on probability proportional to enrollment size from the Shanxi Province. All eligible residents (≥65) were invited to participate in this study. Temporary residents (not listed in the registry office census or living in the selected villages for less than 5 years), those suffering from a life-threatening illness, those who moved to other areas, and those who had passed away were excluded. 

Ethical approval was gained from the Ethics Committee of Health Science Center, Xi’an Jiaotong University and the local hospitals (project number: 2016-221). All participants took part voluntarily, and written informed consent was obtained from each participant before data collection.

### 2.2. Study Procedures

The Chinese government provides free annual health assessment for the elderly aged 65 years and over, which is organized by the local community health center in urban areas and the village clinic in rural areas. In this study, participant recruitment was conducted after the annual health assessment by trained investigators. All investigators were researchers and health professionals with at least 10 years of experience working in public health and were recruited from local township hospitals. Furthermore, a standardized 2-day training program on data collection procedures was provided to all investigators. For residents who were willing to participate in this study, face-to-face interviews were utilized to collect data. 

### 2.3. Instruments

#### 2.3.1. Assessment of FI

For this study, FI was defined as “the involuntary loss of liquid and/or solid stool,” which is accepted by the International Consultation on Incontinence [24]. The self-reported bowel health questionnaire was used to measure FI, which has also presented high levels of reliability regarding continence status [25]. It includes questions from the FI Severity Index, which asks about the frequency of accidental bowel leakage during the past month. Participants reporting any loss of bowel control in the past month were defined as having FI. FI frequency was further assessed as twice or more per day, approximately once per day, twice or more per week, approximately once per week, 1–3 times a month, or never.

#### 2.3.2. Assessment of DI

The concomitant presence of urinary incontinence and FI in the same subject was defined as DI. The Chinese version of International Consultation on Incontinence Questionnaire-Short Form (ICIQ-SF) was used to measure self-reported urinary incontinence within the past 4 weeks [26]. The total score of ICIQ-SF was calculated from three questions, including the frequency (0~5 points) of urine leakage, the severity of urine leakage (0~6 points), and the condition-specific quality of life (0~10 points). The total score ranges from 0 to 21 points, and a total score greater than zero indicates urinary incontinence. 

#### 2.3.3. Associated Factors of FI and DI

All variables selected were based on a comprehensive literature review of previous studies about risk factors contributing to FI or DI and were also reviewed by an expert panel before data collection.

##### Socio-Demographic Assessments

A self-reporting approach was used to collect sociodemographic information. Information was assessed in three key domains: (1) Demographics (sex, age, education, marital status, family income, and medical expenses), (2) lifestyle (regular house/farm work, smoking, alcohol consumption, and habitual tea consumption), and (3) psychosocial factors (living alone or not, sleep quality, social interaction with others in neighborhood, memory complaint, and coping style).

##### Health-Related Factors

Participants’ health-related factors were collected using three approaches, including health records from the local hospital (i.e., relating to hypertension, type 2 diabetes, cerebrovascular disease, heart disease, and hyperlipidemia), interview survey (i.e., hearing impairment, traumatic brain injuries, cognitive function, and activities of daily living (ADL)), and physical examination by trained health professionals (i.e., height, weight, waist circumference, and eyesight). 

Cognitive function, coping style, and ADL were measured by the Chinese vision of the Mini-Mental Status Examination, the 20-item Simplified Coping Style Questionnaire, and the Activities of Daily Living Scale, respectively. 

Mini-Mental Status Examination (MMSE). The MMSE is the most frequently used instrument for cognitive function assessment, with a total score ranging from 0 to 30. It assesses orientation, registration, attention and calculation, recall, and language [27]. Cut-off scores are based on the participants’ education background, with scores ≤17 considered as indicating cognitive impairment for illiteracy, ≤20 for primary school, and ≤24 for secondary school. The Cronbach’s alpha coefficient in this study was 0.722.

Simplified Coping Style Questionnaire (SCSQ). Coping style was measured by the 20-item SCSQ [28,29], which consisted of two dimensions: Positive coping (12 items) and negative coping (8 items). Each item is rated on a four-point Likert scale, where 0 = never, 1 = seldom, 2 = often, and 3 = always. The total score was calculated as the mean difference of positive coping score and negative coping score. If the mean difference was greater than zero, the participant tended to adopt a positive coping style. Otherwise, a negative coping style was employed. The SCSQ has been used widely in Chinese populations and has demonstrated high reliability and validity [28,29]. 

Activities of Daily Living Scale. The ADL scale is a measure that is applied to assess both physical ADL, which includes six items (viz., feeding, dressing, toileting, grooming, ambulating, and bathing) and instrumental ADL (IADL), which consists of eight items (e.g., telephone, shopping, food preparation, etc.) [30]. The Cronbach’s alpha coefficients of the total scale and two subscales were 0.814~0.894 in this study.

### 2.4. Data Analysis 

Data were entered in duplicate using EpiData 3.1 software (the EpiData Association, Odense, Denmark) and then exported to R software (version 3.6.2, R Foundation, Florida, USA) for analysis. Categorical or ordinal variables were expressed as absolute (*n*) and relative (%) frequencies. The prevalence of incontinence with 95% confidence intervals (CIs) were calculated for the overall population. The prevalence differences among subgroups were compared using Pearson’s $\chi^{2}$-test. Univariate logistic regression was performed to select possible associated factors for incontinence (*p* ≤ 0.1). A multivariate logistic regression model with a backward variable selection method was then employed to identify the net effect of factors contributing to incontinence. A two-sided *p* value less than 0.05 was considered statistically significant.

## 3. Results

**Figure 1:**
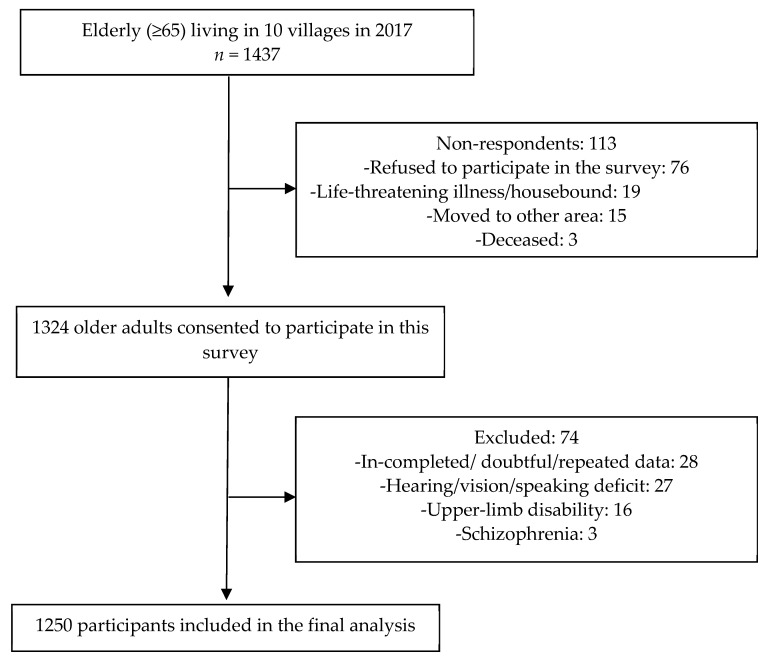
Flow of participants through the study.

In total, 1437 residents were drawn from the 10 selected villages, of which 1324 consented to participate in this survey, yielding a response rate of 92.1%. After excluding 74 ineligible questionnaires, 1250 participants were recruited from these villages (see Figure 1).

### 3.1. Basic Characteristic of Participants

The major characteristics of all participants are presented in Table 1. The mean age was 72.71 years old (SD = 5.36, 65–92) and 56.0% of participants were females. The participants predominantly had a primary school education or less (59.9%), were married (68.9%), regularly engaged in house/farm work (78.6%), and had the government basic living allowance for the rural elderly (80 RMB/month) as their only source of income (50.5%), as shown in Table 1. Hypertension (49.9%) and urinary incontinence (46.6%) were the most common conditions in the participants. A total of 960 (76.8%) participants reported at least one chronic disease, as shown in Table 2. 

### 3.2. Prevalence of FI and DI

FI was reported by 154 of 1250 participants, yielding a prevalence of 12.3% (95% CI 10.5–14.0). Prevalence rates were similar among males (11.8, 95% CI 9.1–14.5) and females (12.7, 95% CI 10.2–15.2). Among all FI cases, the frequency was as follows: Twice or more per day, 12.3% (19/154); approximately once per day, 7.8% (12/154); two or three times per week, 13.0% (20/154); and approximately once per week or less often, 66.9% (103/154; results are not shown in the Table). 

The prevalence of DI was 9.3% (116/1250, 95% CI 7.7–10.9). No sex difference was found in the overall prevalence of DI either. Participants aged 75–79 years demonstrated higher odds of having DI than participants than other age groups (*p* < 0.05). 

### 3.3. Associated Factors of FI and DI

Univariate analyses of sociodemographic, lifestyle, and psychosocial variables are presented in Table 3. Both FI and DI had higher odds of being reported among participants aged 75–79 years, participants having the government basic living allowance as the only income source, participants having higher medical expense, and those reporting poor sleep quality and memory complaints, whereas undertaking regular house or farm work and having good social interaction in the neighborhood were correlated with lower odds (*p* < 0.05). Habitual tea consumption was related to lower odds of having DI (*p* < 0.05).

The univariate analysis of health-related factors is presented in Table 4. Poor hearing, cerebrovascular disease, traumatic brain injury, and ADL dependence, especially physical ADL dependence, were associated with higher odds of having both FI and DI (*p* < 0.05). More chronic diseases and urinary incontinence were found to be correlated with higher odds of having FI (*p* < 0.05).

Multivariate analysis results are presented in Table 5. Multivariate analysis revealed that having the government basic living allowance as the only income source, cerebrovascular disease, traumatic brain injury, and urinary incontinence were significantly associated with higher odds of having FI, whereas having good social interaction in the neighborhood and one chronic disease were associated with lower odds (*p* < 0.05). The overall model for FI was statistically significant, with an explained variance at 22.4% (Nagelkerke R^2^). The odds of having DI were greater in participants with poor sleep quality, traumatic brain injury, and physical ADL dependence, whereas it was lower for people who consumed tea regularly and who had good social interaction with the neighborhood (*p* < 0.05). The model explained 25.2% (Nagelkerke R^2^) of the variation in the outcome of DI. Urinary incontinence (OR 5.17, 95% CI 2.86–9.45) displayed the strongest correlation with FI, whereas physical ADL dependence (OR 9.21, 95% CI 4.47–18.96) demonstrated the strongest correlation with DI.

## 4. Discussion 

This study included a number of crucial findings. First, it confirmed that FI and DI were common in rural residents aged 65 years and older. Second, the prevalence of FI and DI was similar in both sexes, and no significant correlations between age and incontinence prevalence were found in the multivariate analysis. Third, although correlates of FI and DI differed from each other, several shared factors significantly associated with both FI and DI were also found, which provides valuable evidence for individualized interventions and primary care service development. Furthermore, some factors analyzed in this study have not been previously explored among the elderly in rural Asia. 

This study revealed a 12.3% prevalence of FI and a 9.3% prevalence of DI in the rural elderly in China, which is within the range of FI and DI prevalence rates previously reported [2,3,4,5,6,7,8]. However, an epidemiological study by Yuan et al. [18], which investigated FI among 28,196 adult women (≥20 years old) from the urban regions of six provinces and municipalities in China, reported a 9.7% FI prevalence among women aged over 70 years. The possible reason may be geographic difference. In general, the public health conditions and living environment in urban areas are relatively better than those in nonurban areas in China [31]. However, FI prevalence rates are hard to compare across studies due to the lack of a standard definition, as well as differences in populations and investigation methods.

As it has been found in previous studies, the prevalence of FI and DI was similar in males and females [8,10,32]. One possible reason may be that chronic conditions, such as diabetes mellitus, stroke, cognitive impairment, and limited mobility, appear to influence incontinence more strongly than direct pelvic floor injury (e.g., childbirth-related pelvic floor injury for women versus prostate cancer treatment for men) [8,10,32]. However, other researchers have found that DI was significantly more frequent in females than in males [4,33]. It is possible that these studies were based on populations younger than 65 years old. In this younger group, obstetric injuries to the pudendal nerve or sphincter muscle were described as an important risk factor [4,33]. 

Despite the knowledge that aging is a known risk factor for incontinence [18,25], our study failed to confirm these associations, which is consistent with the study conducted in United State adults aged 50 and older [4]. One possible reason for this difference may be differences in populations. Yuan et al. [18] and Ditah et al. [25] investigated FI among people aged 20 years and older in China and the United States, respectively, and found that the odds of having FI were greater in participants aged 55 years and older than those between 20–29 years old. However, neither study compared differences in FI between participants aged 70 years and older. These results may reflect the fact that the prevalence of FI may reach a plateau or peak in older age, or that aging is neither a unique factor in the development of incontinence nor a normal consequence of aging [34]. It remains unclear whether correlations between incontinence and increasing age are directly related or due to functional disabilities (e.g., cognitive impairment and limited mobility) [35]. The relationship between incontinence and aging will be further explored in a longitudinal cohort study. 

Traumatic brain injury was a shared factor associated with high odds of having FI and DI, which was consistent with other studies [36,37]. Two one-year follow-up studies found that traumatic brain injury was associated with an increased risk of urinary incontinence [36] and FI [37]. Traumatic brain injury is a nondegenerative, noncongenital insult to the brain from an external mechanical force, which might result in permanent or temporary impairment of cognitive, physical, or psychosocial function. Incontinence is associated with a poor overall functional outcome following traumatic brain injury [36,37]. These correlations highlight the need for health professionals dealing with incontinence patients to assess whether there is a history of traumatic brain injury. Furthermore, attention should be paid to assessing the urodynamic function of people with traumatic brain injury. 

Both FI and DI were significantly associated with poor social interaction in the neighborhood, as other studies have demonstrated [33,38]. Nakanishi and associates reported that a lack of participation in social activities was significantly associated with DI [38]. An epidemiological study in Brazil found that changes in the habit of going out were significantly associated with higher odds of having DI [33]. Moreover, researchers revealed that individuals with incontinence reported feelings of social isolation from family and friends and reduced their social activities to hide their incontinence [39,40]. Peer support might be an effective intervention, as it increases social interaction for the incontinence population [41]. 

Urinary incontinence was strongly associated with FI, with odds greater than five-fold in the elderly and with more than three-quarters of older adults with FI reporting urinary incontinence in this study. This correlation has been reported in other studies [7,42]. Fecal and urinary incontinence are believed to share etiological factors in women, some of which may include damage to the pelvic floor sustained through childbirth or surgery (hysterectomy) [7,42]. Comprehensive interventions targeting high-risk populations with these shared risk factors for both FI and UI should be developed and tested in primary care settings, which might be a cost-effective strategy for this population.

In our study, physical ADL dependence was the strongest associated modifiable factor, with people suffering from it displaying more than nine-times as great a likelihood of reporting DI. This association was more robust than findings observed in the National Health and Nutrition Examination Survey (2005–2010) in United States, in which ADL dependence was associated with 2.40-times greater odds of having DI [4]. It is believed that the association between physical ADL dependence and incontinence is bidirectional. The impaired ability to undertake ADL may play an essential role in the development of DI, because individuals with limited mobility require more time to perform toileting-related activities [4,5,43,44]. In addition, incontinence may precede physical ADL dependence since incontinence can lead to social isolation, which is an important risk factor for limited mobility [40,45]. Research has demonstrated that both DI and functional dependence share common risk factors, such as more medical comorbidities, predisposing older adults to both conditions [43,44,46]. Environmental interventions such as improved toileting assistance might be of significant benefit to this population. 

This study also revealed that habitual tea consumption was significantly associated with lower odds of having DI. A study from Japan found a similar association between tea drinking (presumably green tea) and urinary incontinence (OR 0.37, 95%CI 0.15–0.91) [47], while another cross-sectional study conducted in Sweden suggested that high tea consumption was associated with high odds of having overactive bladder (OR 1.34, 95%CI 1.07–1.67) and nocturnal enuresis (OR 1.18, 95%CI 1.01–1.38) [48]. Unlike Western countries, the common method of preparing tea by Asian counties is to brew dry tea leaves in a teapot using hot water without adding milk and sugar. The following mechanisms might help to understand the differences: Tea, especially green tea, contains some vitamins and minerals which have an inhibitory effect on urinary stone formation and can reduce glucose levels and renal injury associated with abnormal glucose-related oxidative stress in diabetic nephropathy, while the addition of milk to tea inhibits the antioxidant effects of tea [49,50,51]. The potential effect of tea consumption against DI opens a new avenue for preventing or postponing the onset of DI, as it is an acceptable and economical approach that does not significantly affect other dietary habits for people in rural areas. Longitudinal population-based studies and randomized controlled trials are encouraged for future investigations into the frequency of tea drinking, the type of tea, and other confounding factors. 

We found an interesting association between chronic diseases and FI. Older adults with a chronic disease demonstrated lower odds of having FI than those without chronic disease. One possible reason for this difference may be that residents with chronic diseases, such as hypertension or diabetes, receive more free primary health care services than healthy elderly in China, which is funded by the government [52]. This might also reflect the fact that improved management of chronic disease and screening for potential sequelae of chronic disease might reduce the likelihood or impact of impairment arising from disease [53]. The results might also be associated with potential confounders that were not included in the current study. 

In our study, cognitive impairment was not a significant factor for either FI or DI, which is inconsistent with other studies [54,55,56]. Possible reasons might be that these studies investigated the combined effects of cognitive and functional impairment on incontinence [54,55,56] and were thus unable to determine the effects of cognitive impairment alone on incontinence [57]. The main reasons for accompanying symptoms of incontinence among severely cognitive impaired patients are the inability to find or use the toilet in a timely manner, inappropriately seeking help, and physical impairment [58,59]. However, due to the existence of self-reported data collection methods, no residents with severe cognitive impairment were recruited in this study. This may also explain the absence of a relationship between cognitive impairment and incontinence in our research.

More than 90% of the villages have standard government-funded clinics in rural areas of China, and the elderly mainly receive medical services from rural clinics [60]. Rural health care professionals (HCPs) are the most predominant HCP to manage incontinence in these areas. However, many HCPs lack knowledge of risk factors, symptoms, prevention, treatment, and incontinence management strategies [61]. This lack of knowledge is an important barrier to the implementation of effective incontinence treatments [62]. Considering the high prevalence rates and low consultation rates of incontinence, targeted education for rural HCPs, screening tools, and guidelines applicable to rural areas are needed. 

The study has several limitations. First, the cross-sectional design of our study prevented determination of the causal relationships between incontinence and associated factors. Second, although many factors were included in our study, there could be other unobserved confounding factors that we neither considered nor controlled. Finally, all participants were from one province and the results cannot be generalized to the entire older population in China. 

## 5. Conclusions

This study revealed that FI and DI were prevalent among older adults living in rural China. Correlates, including physical ADL dependence, habitual tea consumption, and social interaction with the neighborhood, are potentially amenable to interventions to improve DI. The estimates of the incontinence prevalence rate and its associated factors in our study might contribute to the increased awareness that there is a need to develop public health policies and primary and secondary prevention programs. Further studies into the effectiveness of conservative management addressing modifiable risk factors in community-dwelling older adults are clearly needed. 

## Figures and Tables

**Table 1:** Major characteristics of study population (*n* = 1250).

Characteristics		Total *N (*%)	FI			DI		
			No FI *N (*%)	FI *N (*%)	*p* Value	No DI *N (*%)	DI *N (*%)	*p* Value
Participants		1250 (100.0)	1096 (87.7)	154 (12.3)	-	1134 (90.7)	116 (9.3)	-
**Demographics**								
Sex	Male	550 (44.0)	485 (44.3)	65 (42.2)	0.632	507 (44.7)	43 (37.1)	0.114
	Female	700 (56.0)	611 (55.7)	89 (57.8)		627 (55.3)	73 (62.9)	
Age (years)	65~69	433 (34.6)	386 (35.2)	47 (30.5)	0.077	401 (35.4)	32 (27.6)	0.011
	70~74	393 (31.4)	346 (31.6)	47 (30.5)		354 (31.2)	39 (33.6)	
	75~79	261 (20.9)	217 (19.8)	44 (28.6)		225 (19.8)	36 (31.0)	
	80~	163 (13.0)	147 (13.4)	16 (10.4)		154 (13.6)	9 (7.8)	
Education	Illiteracy	135 (10.8)	121 (11.0)	14 (9.1)	0.203	125 (11.0)	10 (8.6)	0.209
	Primary school	614 (49.1)	528 (48.2)	86 (55.8)		548 (48.3)	66 (56.9)	
	≥Secondary school	501(40.1)	447 (40.8)	54 (35.1)		461 (40.7)	40 (34.5)	
Marital status	Married	861 (68.9)	752 (68.6)	109 (70.8)	0.587	778 (68.6)	83 (71.6)	0.514
	Divorced/Widowed	389 (31.1)	344 (31.4)	45 (29.2)		356 (31.4)	33 (28.4)	
Income	Subsidies ^※^	631 (50.5)	540 (49.3)	91 (59.1)	0.002	565 (49.8)	66 (56.9)	0.003
	Poverty	260 (20.8)	223 (20.3)	37 (24.0)		228 (20.1)	32 (27.6)	
	Nonpoverty	359 (28.7)	333 (30.4)	26 (16.9)		341 (30.1)	18 (15.5)	
Medical expenses	<Average	814 (65.1)	728 (66.4)	86 (55.8)	0.010	751 (66.2)	63 (54.3)	0.010
	≥Average	436 (34.9)	368 (33.6)	68 (44.2)		383 (33.8)	53 (45.7)	
**Lifestyle**								
Regular house/farm work		886 (70.9)	791 (72.2)	95 (61.7)	0.007	813 (71.7)	73 (62.9)	0.048
Smoking		244 (19.6)	218 (19.9)	26 (17.2)	0.435	227 (20.1)	17 (14.9)	0.187
Alcohol consumption	Never	1106 (88.8)	969 (88.5)	137 (90.7)	0.508	1000 (88.3)	106 (93.0)	0.208
	Sometimes	95 (7.6)	87 (7.9)	8 (5.3)		91 (8.0)	4 (3.5)	
	Often	45 (3.6)	39 (3.6)	6 (4.0)		41 (3.6)	4 (3.5)	
Tea consumption		205 (16.4)	188 (17.2)	17 (11.0)	0.055	197 (17.4)	8 (6.9)	0.004
**Psychosocial factors**								
Living alone		139 (11.1)	125 (11.4)	14 (9.1)	0.392	129 (11.4)	10 (8.6)	0.369
Sleep quality	Good	791 (63.3)	707 (64.5)	84 (54.5)	0.041	733 (64.6)	58 (50.0)	0.005
	Moderate	306 (24.5)	262 (23.9)	44 (28.6)		270 (23.8)	36 (31.0)	
	Poor	153 (12.2)	127 (11.6)	26 (16.9)		131 (11.6)	22 (19.0)	
Social interaction in neighborhood	Good	411 (32.9)	390 (35.6)	21 (13.6)	<0.001	392 (34.6)	19 (16.4)	<0.001
	Satisfactory	804 (64.3)	676 (61.7)	128 (83.1)		709 (62.5)	95 (81.9)	
	Poor	35 (2.8)	30 (2.7)	5 (3.2)		33 (2.9)	2 (1.7)	
Memory complaint		1034 (82.7)	893 (81.5)	141 (91.6)	0.002	925 (81.6)	109 (94.0)	0.001
Coping	Negative	125 (10.0)	104 (9.5)	21 (13.6)	0.108	109 (9.6)	16 (13.8)	0.153
	Positive	1125 (90.0)	992 (90.5)	133 (86.4)		1025 (90.4)	100 (86.2)	

Abbreviations: FI, Fecal Incontinence; DI, DualDouble Incontinence; MMSE, Mini-Mental Status Examination. ※ Basic living allowance provided by the government for rural elderly, RMB 80 Yuan/month (around USD 11.5).

**Table 2:** Health-related characteristics of study population (*n* = 1250).

Characteristics		Total *N (*%)	FI			DI		
			No FI *N (*%)	FI *N (*%)	*p* Value	No DI *N (*%)	DI *N (*%)	*p* Value
BMI (kg/m^2^)	Underweight	90 (7.2)	75 (6.8)	15 (9.7)	0.427	78 (6.9)	12 (10.3)	0.388
	Normal	605 (48.4)	533 (48.6)	72 (46.8)		551 (48.6)	54 (46.6)	
	Overweight	555 (44.4)	488 (44.5)	67 (43.5)		505 (44.5)	50 (43.1)	
Waist circumference (cm)	Normal	509 (40.7)	446 (40.7)	63 (40.9)	0.959	459 (40.5)	50 (43.1)	0.583
	High	741 (59.3)	650 (59.3)	91 (59.1)		675 (59.5)	66 (56.9)	
Hearing	Good	351 (28.1)	323 (29.5)	28 (18.2)	0.003	332 (29.3)	19 (16.4)	0.003
	Moderate	505 (40.4)	443 (40.4)	62 (40.3)		458 (40.4)	47 (40.5)	
	Poor	394 (31.5)	330 (30.1)	64 (41.6)		344 (30.3)	50 (43.1)	
Weak Vision		105 (8.4)	89 (8.1)	16 (10.4)	0.342	91 (8.0)	14 (12.1)	0.135
Chronic disease	0	290 (23.2)	259 (23.6)	31 (20.1)	0.005	266 (23.5)	24 (20.7)	0.071
	1	464 (37.1)	422 (38.5)	42 (27.3)		431 (38.0)	33 (28.4)	
	2	318 (25.4)	268 (24.5)	50 (32.5)		281 (24.8)	37 (31.9)	
	≥3	178 (14.2)	147 (13.4)	31 (20.1)		156 (13.8)	22 (19.0)	
Hypertension		613 (49.0)	527 (48.1)	86 (55.8)	0.071	554 (48.9)	59 (50.9)	0.680
Diabetes		105 (8.4)	91 (8.3)	14 (9.1)	0.741	95 (8.4)	10 (8.6)	0.928
Cerebrovascular disease		94 (7.5)	68 (6.2)	26 (16.9)	<0.001	74 (6.5)	20 (17.2)	<0.001
Heart disease		327 (26.2)	284 (25.9)	43 (27.9)	0.595	291 (25.7)	36 (31.0)	0.210
Hyperlipidemia		140 (11.2)	125 (11.4)	15 (9.7)	0.540	129 (11.4)	11 (9.5)	0.538
Traumatic brain injury		97 (7.8)	66 (6.0)	31 (20.1)	<0.001	74 (6.5)	23 (19.8)	<0.001
Chronic constipation		385 (30.8)	333 (30.4)	52 (33.8)	0.395	342 (30.2)	43 (37.1)	0.125
Urinary incontinence		582 (46.6)	466 (42.5)	116 (75.3)	<0.001	-	-	-
ADL dependence		89 (7.1)	70 (6.4)	19 (12.3)	0.007	73(6.4)	16 (13.8)	0.003
Physical ADL dependence		705 (56.4)	588 (53.6)	117 (76.0)	<0.001	598 (52.7)	107 (92.2)	<0.001
IADL dependence		409 (32.7)	351 (32.0)	58 (37.7)	0.168	364 (32.1)	45(38.8)	0.143
Cognitive impairment		536 (42.9)	463 (42.2)	73 (47.4)	0.226	476 (42.0)	60 (51.7)	0.043

Abbreviations: FI, Fecal Incontinence; DI, Double Incontinence; BMI, Body Mass Index; ADL, Activities of Daily Living.

**Table 3:** Prevalence of FI and DI and univariate analysis.

Characteristics		FI		DI	
		Prevalence (95%CI)	OR (95%CI)	Prevalence (95%CI)	OR (95%CI)
Total		12.3 (10.5, 14.0)		9.3 (7.7, 10.9)	
Sex	Male (ref.)	11.8 (9.1, 14.5)		7.8 (5.6, 10.0)	
	Female	12.7 (10.2, 15.2)	1.09 (0.77–1.53)	10.4 (8.0, 12.8)	1.37 (0.93–2.04)
Age (years)	65~69 (ref.)	10.9 (8.0, 13.8)		7.4 (4.9, 9.9)	
	70~74	12 (8.9, 15.1)	1.12 (0.73–1.71)	9.9 (7.0, 12.8)	1.38 (0.85–2.25)
	75~79	16.9 (12.4, 21.4)	1.67 (1.07–2.60)	13.8 (9.7, 17.9)	2.01 (1.21–3.32)
	80~	9.8 (5.3, 14.3)	0.89 (0.49–1.63)	5.5 (2.0, 9.0)	0.73 (0.34–1.57)
Education	Illiteracy (ref.)	10.4 (5.3, 15.5)		7.4 (2.9, 11.9)	
	Primary school	14 (11.3, 16.7)	1.41 (0.77–2.56)	10.7 (8.3, 13.1)	1.51 (0.75–3.01)
	≥Secondary school	10.8 (8.1, 13.5)	1.04 (0.56–1.94)	8 (5.6, 10.4)	1.09 (0.53–2.23)
Marital status	Married (ref.)	11.6 (8.5, 14.7)		9.6 (7.6, 11.6)	
	Divorced/Widowed	12.3 (10.5, 14.1)	0.90 (0.62–1.31)	8.5 (5.8, 11.2)	0.87 (0.57–1.33)
Income	Subsidies (ref.) ^※^	14.4 (11.7, 17.1)		10.5 (8.1, 12.9)	
	Poverty	14.2 (9.9, 18.5)	0.99 (0.65–1.49)	12.3 (8.4, 16.2)	1.20 (0.76–1.88)
	Nonpoverty	7.2 (4.5, 9.9)	0.46 (0.29–0.73)	5 (2.6, 7.4)	0.45 (0.26–0.77)
Medical expenses	<Average (ref.)	10.6 (8.4, 12.8)		7.7 (5.9, 9.5)	
	≥Average	15.6 (12.3, 18.9)	1.56 (1.11–2.20)	12.2 (9.1, 15.3)	1.65 (1.12–2.43)
Regular house/farm work	No (ref.)	16.2 (12.5, 19.9)		11.8 (8.5, 15.1)	
	Yes	10.7 (8.7, 12.7)	0.62 (0.44–0.88)	8.2 (6.4, 10.0)	0.67 (0.45–0.99)
Smoking	No (ref.)	12.5 (10.5, 14.5)		9.7 (7.9, 11.5)	
	Yes	10.7 (6.8, 14.6)	0.84 (0.54–1.31)	7 (3.9, 10.1)	0.70 (0.41–1.19)
Alcohol consumption	Neve r(ref.)	12.4 (10.4, 14.4)		9.6 (7.8, 11.4)	
	Sometimes	8.4 (2.9, 13.9)	0.65 (0.31–1.37)	4.2 (0.1, 8.3)	0.42 (0.15–1.15)
	Often	13.3 (3.3, 23.3)	1.09 (0.45–2.62)	8.9 (0.7, 17.1)	0.92 (0.32–2.62)
Tea consumption	No (ref.)	13.1 (11.1, 15.1)		10.3 (8.5, 12.1)	
	Yes	8.3 (4.6, 12.0)	0.60 (0.35–1.02)	3.9 (1.2, 6.6)	0.35 (0.17–0.73)
Living alone	No (ref.)	12.6 (10.6, 14.6)		9.5 (7.7, 11.3)	
	Yes	10.1 (5.0, 15.2)	1.29 (0.72–2.30)	7.2 (2.9, 11.5)	1.36 (0.69–2.67)
Sleep quality	Good (ref.)	10.6 (8.4, 12.8)		7. 3 (5.5, 9.1)	
	Moderate	14.4 (10.5, 18.3)	1.41 (0.96–2.09)	11.8 (8.3, 15.3)	1.69 (1.09–2.61)
	Poor	17 (11.1, 22.9)	1.72 (1.07–2.78)	14.4 (8.9, 19.9)	2.12 (1.26–3.59)
Social interaction in neighborhood	Good (ref.)	5.1 (2.9, 7.3)		4.6 (2.6, 6.6)	
	Satisfactory	15.9 (13.4, 18.4)	3.52 (2.18–5.67)	11.8 (9.6, 14.0)	2.76 (1.66–4.59)
	Poor	14.3 (2.7, 25.9)	3.10 (1.09–8.79)	5.7 (0.0, 13.3)	1.25 (0.28–5.60)
Memory complaint	No (ref.)	6 (2.9, 9.1)		3.2 (0.8, 5.6)	
	Yes	13.6 (11.4, 15.8)	2.47 (1.37–4.44)	10.5 (8.5, 12.5)	3.52 (1.62–7.67)
Coping	Negative (ref.)	16.8 (10.3, 23.3)		12.8 (6.9, 18.7)	
	Positive	11.8 (9.8, 13.8)	0.66 (0.40–1.10)	8.9 (7.3, 10.5)	0.67 (0.38–1.17)

Abbreviations: FI, Fecal Incontinence; DI, Double Incontinence. ^※^ Basic living allowance provided by the government for rural elderly, RMB 80 Yuan/month (around USD 11.5).

**Table 4:** Prevalence of FI and DI and univariate analysis of health-related factors.

Characteristics		FI		DI	
		Prevalence (95%CI)	OR (95%CI)	Prevalence (95%CI)	OR (95%CI)
BMI (kg/m^2^)	Underweight (ref.)	12.3 (10.5, 14.1)		13.3 (6.2, 20.4)	
	Normal	16.7 (9.1, 24.3)	0.68 (0.37–1.24)	8.9 (6.5, 11.3)	0.64 (0.33–1.24)
	Overweight	11.9 (9.4, 14.4)	0.69 (0.37–1.26)	9 (6.6, 11.4)	0.64 (0.33–1.26)
Waist circumference (cm)	Normal (ref.)	12.4 (9.5, 15.3)		9.3 (7.7, 10.9)	
	High	12.3 (9.9, 14.7)	0.99 (0.70–1.40)	9.8 (7.3, 12.3)	0.90 (0.61–1.32)
Hearing	Good (ref.)	8 (5.3, 10.7)		5.4 (3.0, 7.8)	
	Moderate	12.3 (9.4, 15.2)	1.61 (1.01–2.58)	9.3 (6.8, 11.8)	1.79 (1.03–3.11)
	Poor	16.2 (12.5, 19.9)	2.24 (1.4–3.58)	12.7 (9.4, 16.0)	2.54 (1.47–4.40)
Vision	Normal	12.1 (10.1, 14.1)		8.9 (7.3, 10.5)	
	Weak	15.2 (8.3, 22.1)	1.31 (0.75–2.30)	13.3 (6.8, 19.8)	1.57 (0.87–2.86)
Chronic disease	0 (ref.)	10.7 (7.2, 14.2)		8.3 (5.2, 11.4)	
	1	9.1 (6.6, 11.6)	0.83 (0.51–1.36)	7.1 (4.7, 9.5)	0.85 (0.49–1.47)
	2	15.7 (11.8, 19.6)	1.56 (0.97–2.52)	11.6 (8.1, 15.1)	1.46 (0.85–2.51)
	≥3	17.4 (11.9, 22.9)	1.76 (1.03–3.02)	12.4 (7.5, 17.3)	1.56 (0.85–2.88)
Hypertension	No (ref.)	10.7 (8.3, 13.1)		8.9 (6.7, 11.1)	
	Yes	14 (11.3, 16.7)	1.37 (0.97–1.92)	9.6 (7.2, 12.0)	1.08 (0.74–1.59)
Diabetes	No (ref.)	12.2 (10.2, 14.2)		9.3 (7.5, 11.1)	
	Yes	13.3 (6.8, 19.8)	1.10 (0.61–1.99)	9.5(3.8, 15.2)	1.03 (0.52–2.04)
Cerebrovascular disease	No (ref.)	11.1 (9.3, 12.9)		8.3 (6.7, 9.9)	
	Yes	27.7 (18.7, 36.7)	3.07 (1.89–5.00)	21.3 (13.1, 29.5)	2.98 (1.75–5.10)
Heart disease	No (ref.)	12 (9.8, 14.2)		8.7 (6.9, 10.5)	
	Yes	13.1 (9.4, 16.8)	1.11 (0.76–1.62)	11 (7.7, 14.3)	1.30 (0.86–1.98)
Hyperlipidemia	No (ref.)	10.7 (8.3, 13.1)		9.5 (7.7, 11.3)	
	Yes	14 (11.3, 16.7)	0.84 (0.48–1.47)	7.9 (3.4, 12.4)	0.82 (0.43–1.56)
Traumatic brain injury	No (ref.)	10.7 (8.9, 12.5)		8.1 (6.5, 9.7)	
	Yes	32(22.8, 41.2)	3.93 (2.47–6.27)	23.7 (15.3, 32.1)	3.54 (2.12–5.92)
Chronic constipation	No (ref.)	11.8 (9.6, 14.0)		8.4 (6.6, 10.2)	
	Yes	13.5 (10.2, 16.8)	1.17 (0.82–1.67)	11.2 (8.1, 14.3)	1.36 (0.92–2.03)
Urinary incontinence	No (ref.)	5.7 (3.9, 7.5)		-	
	Yes	19.9 (16.6, 23.2)	4.13 (2.81–6.07)	-	-
ADL dependence	No (ref.)	11.6 (9.8, 13.4)		8.6 (7.0, 10.2)	
	Yes	21.3 (12.9, 29.7)	2.06 (1.21–3.53)	18 (10.0, 26.0)	2.33 (1.30–4.15)
Physical ADL dependence	No (ref.)	6.8 (4.6, 9.0)		1.7 (0.7, 2.7)	
	Yes	16.6 (13.9, 19.3)	2.73 (1.85–4.03)	15.2 (12.5, 17.9)	10.66 (5.35–21.25)
IADL dependence	No (ref.)	11.4 (9.2, 13.6)		8.4 (6.4, 10.4)	
	Yes	14.2 (10.9, 17.5)	1.28 (0.90–1.82)	11 (8.1, 13.9)	1.34 (0.90–1.99)
Cognitive impairment (MMSE)	No (ref.)	11.3 (8.9, 13.7)		7.8 (5.8, 9.8)	
	Yes	13.6 (10.7, 16.5)	1.23 (0.88–1.73)	11.2 (8.5, 13.9)	1.48 (1.01–2.17)

Abbreviations: FI, Fecal Incontinence; DI, Double Incontinence; BMI, Body Mass Index; ADL, Activities of Daily Living.

**Table 5:** Multivariate analysis of factors associated with FI and DI.

Variables		FI	DI
		OR (95%CI)	OR (95%CI)
Age (years)	65~69 (ref.)		
	70~74	0.74 (0.46–1.20)	0.93 (0.54–1.61)
	75~79	1.04 (0.62–1.75)	1.25 (0.70–2.25)
	80~	0.52 (0.26–1.05)	0.43 (0.18–1.01)
Female sex		0.75 (0.49–1.16)	0.82 (0.51–1.34)
Income	Subsidies (ref.) ^※^		
	Poverty	0.92 (0.57–1.49)	1.22 (0.72–2.08)
	Nonpoverty	0.52 (0.31–0.87)	0.56 (0.31–1.02)
Medical expenses (≥average vs. < average)		1.21 (0.81–1.80)	1.27 (0.81–1.98)
Regular house/farm work (yes vs. no)		0.77 (0.48–1.22)	1.02 (0.61–1.73)
Tea consumption (yes vs. no)		0.76 (0.42–1.37)	0.44 (0.19–0.98)
Sleep quality	Good (ref.)		
	Moderate	1.21 (0.79–1.87)	1.49 (0.92–2.41)
	Poor	1.44 (0.85–2.47)	1.83 (1.02–3.28)
Social interaction in neighborhood	Good (ref.)		
	Satisfactory	3.73(2.24–6.20)	2.95 (1.70–5.13)
	Poor	2.69(0.87–8.26)	0.91 (0.17–4.79)
Memory complaint (yes vs. no)		1.53 (0.79–2.96)	2.24 (0.96–5.25)
Cognitive impairment (MMSE) (yes vs. no)		-	1.00 (0.64–1.55)
Hearing	Good (ref.)		
	Moderate	1.00(0.59–1.61)	1.07 (0.58–1.98)
	Poor	1.37 (0.78–2.38)	1.36 (0.72–2.58)
Chronic disease	0 (ref.)		
	1	0.54 (0.30–0.97)	0.58 (0.32–1.05)
	2	0.62 (0.31–1.23)	0.72 (0.39–1.35)
	≥3	0.46 (0.20–1.09)	0.50 (0.23–1.10)
Hypertension (yes vs. no)		1.55 (0.94–2.55)	-
Cerebrovascular disease (yes vs. no)		1.86 (1.00–3.47)	1.77 (0.90–3.48)
Traumatic brain injury (yes vs. no)		2.96 (1.62–5.42)	2.80 (1.44–5.46)
Urinary incontinence (yes vs. no)		5.17 (2.86–9.45)	-
ADL dependence (yes vs. no)		1.15 (0.58–2.26)	0.88 (0.44–1.76)
Physical ADL dependence (yes vs. no)		0.70 (0.38–1.32)	9.21 (4.47–18.96)

Abbreviations: FI, Fecal Incontinence; DI, Double Incontinence; ADL, Activities of Daily Living. ^※^ Basic living allowance provided by the government for rural elderly, RMB 80 Yuan/month (around USD 11.5).
